# A Novel Microfluidic Dielectrophoresis Technology to Enable Rapid Diagnosis of *Mycobacteria tuberculosis* in Clinical Samples

**DOI:** 10.1016/j.jmoldx.2023.04.005

**Published:** 2023-06-22

**Authors:** Catherine M. Moore, Jasvir Dhillon, Rebecca Flynn, Krzysztof Gizynski, Candice Adams, George Morgan, David McGurk, Eduardo Boada, Shireen Shabestary, Jonathan Peat, Jonathan O'Halloran, Neil G. Stoker, Philip D. Butcher, Heather Murton

**Affiliations:** ∗ Institute for Infection and Immunity, St. George's University of London, London, United Kingdom; † QuantuMDx Group Ltd., Newcastle upon Tyne, United Kingdom

## Abstract

To achieve the global efforts to end tuberculosis, affordable diagnostics suitable for true point-of-care implementation are required to reach the missing millions. In addition, diagnostics with increased sensitivity and expanded drug susceptibility testing are needed to address drug resistance and to diagnose low-bacterial burden cases. The laboratory-on-a-chip technology described herein used dielectrophoresis to selectively isolate *Mycobacterium tuberculosis* from sputum samples, purifying the bacterial population ahead of molecular confirmation by multiplex real-time quantitative PCR. After optimization using a panel of 50 characterized sputum samples, the performance of the prototype was assessed against the current gold standards, screening 100 blinded sputum samples using characterized and biobanked sputum provided by Foundation for Innovative New Diagnostics. Concordance with culture diagnosis was 100% for smear-negative samples and 87% for smear-positive samples. Of the smear-positive samples, the high burden sample concordance was 100%. Samples were diagnosed on the basis of visual assessment of the dielectrophoresis array and by multiplex real-time quantitative PCR assay. The results described herein demonstrate the potential of the CAPTURE-XT technology to provide a powerful sample preparation tool that could function as a front-end platform for molecular detection. This versatile tool could equally be applied as a visual detection diagnostic, potentially associated with bacterial identification for low-cost screening or coupled with an expanded PCR assay for genotypic drug susceptibility testing.

Tuberculosis (TB) presents a multifaceted challenge to diagnostics, which impedes treatment access and sustains global transmission.^1^ Pulmonary tuberculosis, the most common presentation of the *Mycobacterium tuberculosis* (MTB) infection in humans, is primarily diagnosed from analysis of sputum samples.^2^ Traditionally, smear microscopy is used with Ziehl-Neelsen staining to specifically highlight acid-fast bacterium.^3^

Mycobacterial burden is measured as grades of smear positivity (3+ to scanty), which is used to evaluate disease severity and associated infectiousness of the patient. Although this method is low cost and requires minimal laboratory facilities, sensitivity is poor, with a detectable limit of 10^4^ bacilli/mL of sputum, and the quality of the diagnostic can vary between site and operators.^4^^,^^5^ More recent improvements to smear microscopy have been implemented, including fluorescent cell labeling with the auramine stain, but lower burden infections still remain undiagnosed.^6^ For higher sensitivity interrogation of so-called smear-negative samples, culture has remained the World Health Organization–recommended method.^7^ The time to culture positivity in liquid culture system has long been used to predict patient outcome. Sputum samples are first decontaminated to eliminate competing, faster growing commensal bacteria and then incubated in growth medium to determine the presence of MTB.^8^ Although undisputedly the most sensitive diagnostic, with limits of detection from 1 to 10 bacilli/mL of sputum, the slow growth rate of MTB and requirement of biosafety level 3 facilities to effectively perform this method of diagnosis remain limitations, particularly in resource-restricted settings.^9^

Meeting the Sustainable Development Goals for Tuberculosis requires the design and implementation of new technologies to address the key bottlenecks in TB control and elimination.^10^ Novel diagnostic technologies are required to improve case detection, thereby reaching the missing millions, estimated in 2019 to be 2.9 million individuals globally.^11^^,^^12^ Cepheid's GeneXpert MTB/RIF assay and the GeneXpert MTB/RIF Ultra (Cepheid, Sunnyvale, CA) is the World Health Organization's main recommended rapid diagnostic test for detection of TB and rifampicin resistance as other more portable technologies are currently not available to provide accurate and rapid diagnosis.^13^ There is still a need for rapid, accurate, and robust TB diagnostic tests suitable for use at the point of care,^14^ with high sensitivity for children and patients with HIV co-infection.

The imperative for these much-needed advances will not just be based on increased sensitivity of detection in new assay formats for pathogen-specific DNA assays, but equally on developing *in vitro* diagnostic platforms applicable to high-endemic, underresourced areas of the world.^15^ This requires devices that are portable and not dependent on infrastructure, yet suitable for use with difficult to manipulate sputum samples and capable of providing purified samples to test, as well as enhanced sensitivity of detection.^16^

Herein we describe a prototype microfluidic chip-based system that can process solubilized sputum from patients with suspected TB, capture MTB bacilli for visual analysis (as a substitute for smear microscopy), and provide a purified sample for molecular confirmation by real-time quantitative PCR (qPCR) and ultimately for drug-susceptibility analysis.

CAPTURE-XT technology (QuantuMDx Group Ltd., Newcastsle, UK) employs dielectrophoresis (DEP) separation of MTB bacilli from the patient sample, allowing isolation and differential purification of the bacilli from other organisms and impurities that can mask detection or interfere with molecular analysis.^17^ DEP exploits the relative polarizability of a particle and the medium in which it exists to manipulate the particle's movement. The DEP force is governed by properties of the particle of interest and the medium in which it exists (sample or buffer) such that a capture field can be generated by applying a voltage to an array of electrodes interfaced with the solution in which the particles are suspended. Since the system was first described, DEP has been the subject of numerous studies and coupled with microfluidics for application as biosensors and environmental sensors and in the development of medical diagnostics. Such work has been extensively covered in reviews.^18^, ^19^, ^20^

DEP therefore represents a novel and as yet untested approach to TB diagnosis that could provide a test with improved performance for low bacterial burden sputum samples, such as smear scanty or smear-negative (but culture-positive) samples—an area of current diagnostic inaccuracy and low performance. A prototype microfluidic DEP purifier was used here ahead of a qPCR assay to assess DEP performance against gold standard TB diagnostics on a blinded set of 100 human sputum samples provided by the Foundation for Innovative New Diagnostics (FIND).

## Materials and Methods

### Samples

Aliquots (0.2 to 0.5 mL) of human sputum were provided by FIND from a biobank repository (ZeptoMetrix Corp., Franklin, MA) with ethical approval from 100 patients. Samples were previously comprehensively characterized, and relevant patient history was recorded. Gold standard diagnostics were used for determination of the patients' disease status, including at least liquid/solid culture and smear microscopy (Ziehl-Nielsen).^21^ Sputum samples originated from South Africa, Peru, and Vietnam and were provided blinded with a 12-digit unique identifier. Sputum samples were stored at −20°C until required for analysis. An additional 50 samples were provided with known smear and culture status for use in protocol optimization. The 50-sample panel consisted of 20 negative samples (smear negative; culture negative); 9 scanty samples (smear negative; culture positive); and 21 positive samples (smear positive graded between 1+ to 3+; culture positive). The blinded data set was predominantly made up of negatives and scanty/smear-negative, culture-positive samples by request, as these are the most challenging samples to confirm as positive for MTB for any TB diagnostic.

### Sample Processing Protocol

A 500-μL sputum sample was thawed at room temperature for 5 minutes before the addition of 500 μL thinning buffer [2% dithiothreitol (Sigma, Darmstadt, Germany), 1% Tween 80 (Calbiochem, UK; now Merck Life Science UK Limited, Watford, UK), 1% Triton X-100 (Sigma), and 20 mmol/L EDTA, pH 7 (Sigma), in sterile deionized water]. Samples were vortexed for 5 minutes and incubated at 40°C for 90 minutes before addition of 3 μL Baclight cell stain mix (Invitrogen, Waltham, MA). Samples were then incubated at 40°C for a further 30 minutes before the addition of 24 mL sterile deionized water. Samples were vortexed for 5 minutes and filtered through an 8-μm nucleopore filter (Whatman Nucleopore; Merck Life Science UK Limited, Gillingham, UK) and then subjected to probe sonication (Rinco Ultrasonics UK Ltd., London, UK) at 70% amplitude for 25 seconds in 5-second intervals. All samples were processed in a biosafety level 3 laboratory.

### Microfluidic DEP Device and Platform

**Figure 1:**
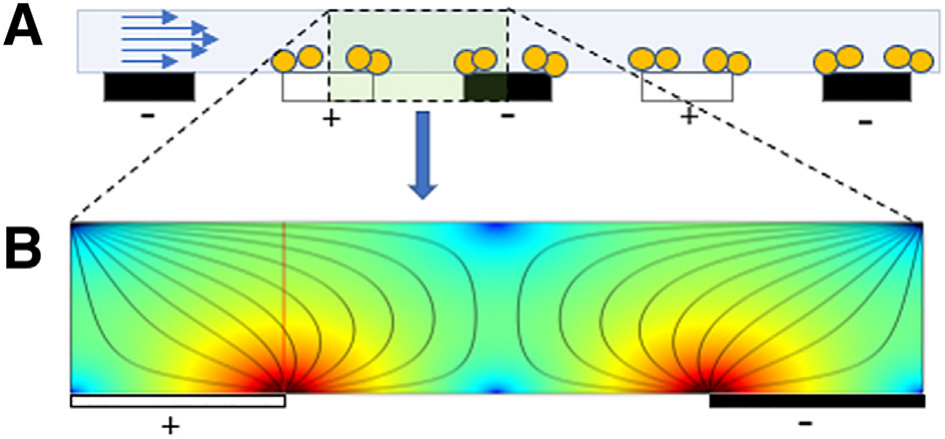
A and B: Schematic of end-on electrodes within the dielectrophoresis (DEP) chip (**A**) and the DEP fields within a microfluidic channel (**B**). **A:** Yellow circles denotes particle of interest (eg, bacteria). **Blue arrows** indicate sample under flow. The interdigitated electrode array has an alternating current applied, shown as black (negative) or white (positive) rectangles. **B: Lines** represent DEP field. Color represents field strength, where red denotes strong attractive forces.

The principles of DEP are based on a phenomenon originally described by Pohl and Hawk^22^ and Pohl and Crane.^23^ Figure 1 shows a schematic of particles (bacteria) attracted to the edges of the electrodes generating the electrical field.

DEP electrodes (Au/Ni) were fabricated onto poly (methyl methacrylate) substrate with microfluid channels defined in SU-8 photoresist (Epigem, Redcar, UK). Fluidics were designed such that eight parallel channels controlled the flow of solution across the electrodes from a single inlet and converged to a single outlet for simple fluid handling. Fluidic connections were made from the microfluidic device using standard connectors (IDEXX) and machined mounts. Devices were flushed with sterile deionized water to void air before sample was introduced. Electronic connections were made by pogo and Bayonet Neill-Concelman (BNC) connectors (RS Components Ltd., Corby, UK) to a function generator (Textronix AFG1022 Arbitrary Waveform Generator; RS Components Ltd.) and signal amplifier (Tabor 9250; Tabor Electronics, Nesher, Israel). A sinusoidal a.c. was established [10 MHz, 1 Vpp (peak-to-peak voltage)] from two channels, connecting to two independently circuited electrode arrays on the DEP device: channel 1 and channel 2.

Two iterations of the DEP control platform were used simultaneously with comparable performance. The first system, built with standard laboratory equipment, utilized a BX53F microscope (Olympus, Tokyo, Japan) with image acquisition and processing via ImageJ software version 1.51 (NIH, Bethesda, MD).^24^ Fluid control was completed via a syringe pump (Cole-Palmer Instrument Co. Ltd., Saint Neots, UK). The second iteration moved toward integration of control components within a bespoke platform. Platform components and imaging systems (ThorLabs Ltd., Ely, UK) alongside pressure-driven fluidics (Fluigent, Le Kremlin-Bicêtre, France) were controlled through a single user interface in μManager software.^25^

In both iterations, filter sets compatible with excitation/emission of 485/498 nm were used to detect the presence or absence of bacilli attracted to the edges of the electrodes, and images were captured using an R3 camera (Retiga; Teledyne Photometrics, Tucson, AZ). Bacilli in the thinned sputum samples were stained with BacLight Bacterial viability stain, following manufacturer's instructions (Thermo Fisher Scientific, Dartford, UK).

**Figure 2:**
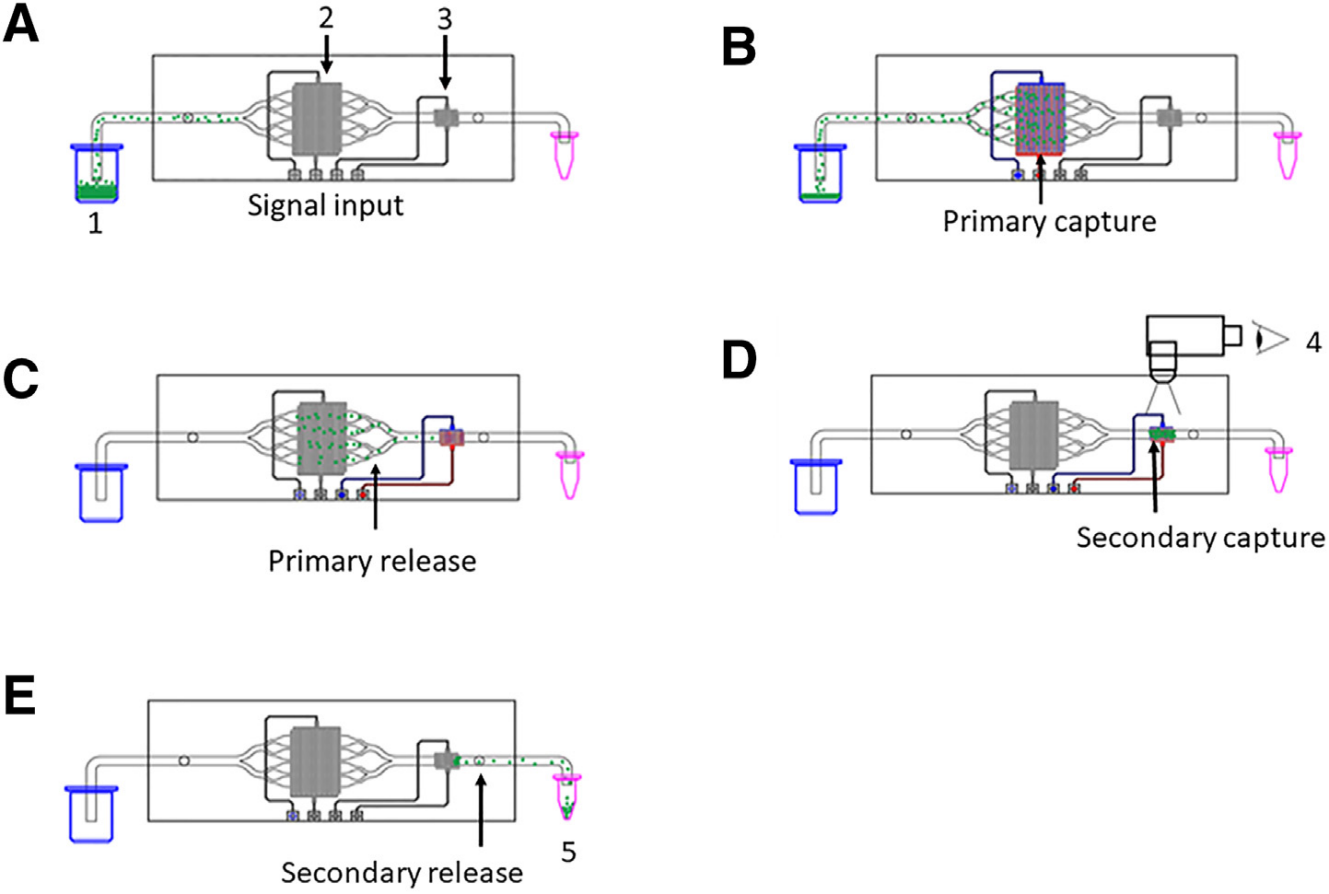
*Mycobacterium tuberculosis* (MTB) isolation process flow: **A:** Sputum sample is thinned, stained, and connected to the fluidic system (1). Sample is directed through the microfluidic cassette, flowing over parallel arrays of interdigitated electrodes (2 and 3). **B:** The primary electrode arrays are activated, establishing a capture field, attracting the MTB toward the primary electrode edges (2). **C:** When the sample is almost exhausted, the smaller, secondary electrode array is activated (3). The primary arrays are deactivated, releasing the bacteria from the capture fields into the flow. **D:** The MTB samples are recaptured on the secondary electrode array, which can be visualized by fluorescent microscopy (4). The presence of fluorescent foci indicates a positive result for tuberculosis infection. **E:** Deactivation of the secondary electrode array releases cells into the flow, allowing collection in a small volume (as low as 5 μL) for molecular analysis (5).

Under DEP, bacilli are attracted to areas of high field density generated by arrays of interdigitated electrodes, powered by an a.c. signal (Figure 1). To increase the processing capacity of the test devices, fluidics were designed to allow dual-phase isolation. For initial capture and volume reduction, the sample was split across eight parallel channels within which the bacilli are collected and immobilized onto primary electrode arrays until the sample has run near to completion. At the end of the sample volume, the DEP fields are de-activated, releasing the bacteria back into the convergent flow streams for secondary capture and concentration onto a single (secondary) electrode bed, where their presence can be confirmed by fluorescence microscopy. Once visual analysis is complete, the DEP field is again de-activated, releasing the bacteria into the flow stream for elution from the microfluidic device. The purified sample can then be used for molecular analysis. The process is outlined graphically in Figure 2.

Processed sputum was loaded onto the syringe pump and flowed through the device to establish a linear flow velocity of 25 to 35 μL/minute across the primary capture fields. The electrode bed was activated (channel 1) with a 10-MHz, 1-Vpp current and remained active until the sample had processed to 90%. Channel 1 was deactivated, terminating the field from the primary capture electrode and, simultaneously, the secondary capture electrode was activated (channel 2). After visual analysis of the secondary electrode (×100 magnification with green fluorescent protein filter), the presence or absence of bacilli was noted and recorded photographically (Retiga R3 camera with μManager software). Where capture/time was measured, the number of bacilli were counted using a countstack plugin in ImageJ software to count particles, compiled over 1 minute.

### Bacteriology: Cultures and Quantitation

For optimization of the system MTB-negative sputum samples spiked with various mycobacterial strains were tested. *Mycobacterium tuberculosis*, strain H37Ra (ATCC, Manassas, VA) and *Mycobacterium smegmatis* MC^2^ 155 (ATCC) were kept as stock strains and stored in liquid nitrogen. These were cultured and serially transferred at weekly intervals (1 mL of 10^8^ seeding culture added to 8-mL broth) in Middlebrook 7H9 (SLS, Nottingham, UK) supplemented with 10% albumin-dextrose-catalase (Becton Dickinson, Franklin Lakes, NJ) and 0.2% glycerol. The cultures were incubated at 37°C. The cultures were serially transferred for 5 weeks before a fresh culture was started.

### Quantitative PCR Assay and Specificity Testing

A triplex quantitative PCR assay using TaqMan probes was established for three specific MTB targets: two established gene targets (*IS6110* and *IS1081*) and *Rv1707*, which was designed in-house. Primers and probes were designed using the Primer3plus program^26^ with emphasis on minimal amplicon size for rapid amplification. *Rv1707* was selected as an MTB-specific locus, whereas the multicopy targets *IS6110* and *IS1081* were included to increase the sensitivity of MTB complex detection. Bioinformatic analysis of the gene target primer pairs and probes by Basic Local Alignment Search Tool was undertaken to confirm nonhomology across species. Primers, probes, melting temperature values, amplicon size, and copy number for each gene target are shown in Table 1. A threshold cycle (C_T_) cutoff value of 38 was assigned on the basis of the GeneXpert manual, which designates any sample with C_T_ value of ≥38 as negative.

**Table 1:** PCR Primers and Probe Sequences Used in Triplex qPCR Assay

Primer	Sequence	Tm, °C	Amplicon size, bp	Copy number∗
*RV1707* F	5′-ATCATCATGACCCAGCTGGATC-3′	63.5	134	1
*RV1707* R	5′-GAGCTGACCAGAACCAGGAC-3′	59.8		
*IS6110* F	5′-CGAACTCAAGGAGCACATCA-3′	60.0	135	16
*IS6110* R	5′-AGTTTGGTCATCAGCCGTTC-3′	60.1		
*IS1081* F	5′-CTGCTCTCGACGTTCATCGCCG-3′	71.6	135	6
*IS1081* R	5′-GGCACGGGTGTCGAAATCACG-3′	70.1		


Probe	Sequence	Tm, °C	Fluorophore	Copy number

*RV1707*	5′-CGTCCTGCTGCTGGCTAGCG-3′	68.9	HEX	
*IS6110*	5′-ACCGTCAGGGCATCGAGGTG-3′	68.0	FAM	
*IS1081*	5′-GCGATGAGCGGTCCAATCAGCGCAA-3′	77.9	Texas red	


F, forward; qPCR, real-time quantitative PCR; R, reverse; Tm, melting tempearture.

∗ Copy number in *Mycobacterium tuberculosis* (MTB) H37Rv. Strains of MTB have variable numbers of *IS6110*, ranging from 0 to 16.

An exclusivity testing panel consisting of nontuberculous mycobacteria and a range of Gram-positive and Gram-negative bacteria was obtained from The Belgian Co-ordinated Collection of Microorganisms (Brussels, Belgium), and from Prof. Tim Bull (St. George's University of London, London, UK). Each primer set was tested against the panel to determine the specificity of the primers. PCR was performed using Qiagen (Hilden, Germany) Multiplex PCR master mix, following the manufacturer's instructions, with 1 ng DNA and 2 μmol/L of each primer and probe per reaction. CFX Real-Time PCR Detection Systems (BioRad, Watford, UK) cycling conditions were 95°C for 15 seconds; followed by 40 cycles of 30 seconds at 94°C, 90 seconds at 57°C, and 60 seconds at 72°C; and finally a 10-minute incubation at 72°C. Each DNA sample was tested both alone and spiked with TB DNA as an internal control. The PCR products were analyzed electrophoretically using the Agilent 2100 Bioanalyzer (Agilent, Santa Clara, CA) (Supplemental Table S1).

For molecular analysis of the CAPTURE-XT eluates, using the *IS6110* qPCR assay, channel 2 was deactivated, terminating the secondary capture field, and a 15-μL eluate was collected from the device outlet into 2-mL screw cap tubes. Several eluates were collected under different conditions, such as during electrode activation, as well as a non-DEP sample, which was thinned sputum only. Device eluates and thinned sputum samples were subject to heat-kill via incubation in a 95°C water bath for 45 minutes to render samples safe to be taken out of the containment level 3 laboratory. Samples were then processed for cell lysis by the addition of 5 μL microlysis solution reagent (Clent Life Sciences, Stourbridge, UK) before heat cycling (80°C for 15 minutes, 96°C for 2 minutes, 80°C for 4 minutes, 96°C for 1 minute, 80°C for 1 minute, and 96°C for 0.5 minutes). A total of 2 μL of eluate was used as template in qPCR, as described with *IS6110* primers and probe.

### Discordance Testing

Following unblinding, samples that had been called incorrectly were labeled discordant and were reprocessed for repeated testing using a second aliquot of sputum. Higher-capacity DEP devices (64 parallel channels rather than 8) were utilized to reduce processing time. Also, to increase specificity, the *IS6110* PCR was supplemented with *IS1081* and an MTB-specific target (*Rv1707*). The third aliquot was subject to culture to detect and confirm the presence of possible low levels of MTB in the sample; this was achieved using selective Kirchner medium with calf serum (SLS) and antibiotic cocktail (polymyxin B, 200,000 U/L; ticarcillin, 100 mg/L; amphotericin B, 10 mg/L; and trimethoprim, 10 mg/L) (Mast Laboratories, Bootle, UK) or Middlebrook 7H9 media [0.2% glycerol and 10% oleic albumin dextrose catalase growth supplement (OADC)] and antibiotic cocktail (polymyxin B, 200,000 U/L; ticarcillin, 100 mg/L; amphotericin B, 10 mg/L; and trimethoprim, 10 mg/L) (Mast Laboratories) at 37°C for 2 to 3 weeks, following which the liquid culture was plated onto Middlebrook 7H11 Agar Plates [10% OADC and 0.5% glycerol plus antibiotic cocktail (polymyxin B, 200,000 U/L; ticarcillin, 100 mg/L; amphotericin B, 10 mg/L; and trimethoprim, 10 mg/L)] (Mast Laboratories), incubated at 37°C for 3 to 4 weeks, and assessed for colony growth.

### Analysis

Statistically significant differences in C_T_ values between thinned sputum samples and post-DEP eluates were estimated using *t*-test, two tailed and paired.

## Results

### CAPTURE-XT Device Optimization

**Figure 3:**
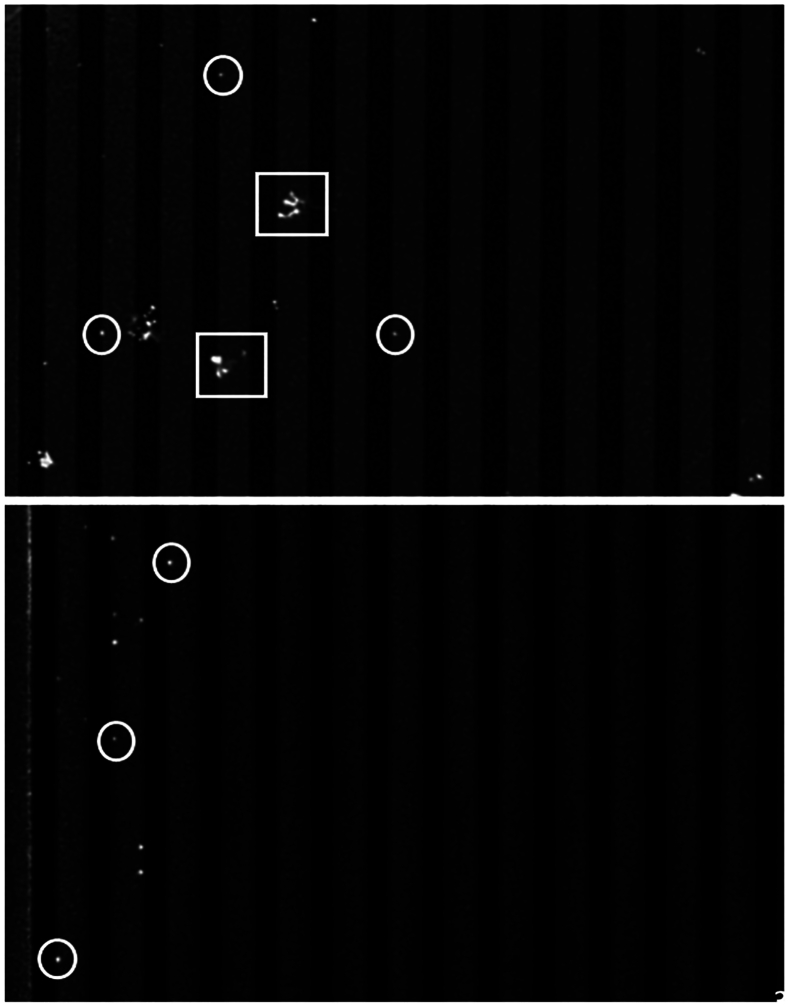
Comparison of sputum versus bacteria. **Top panel:** An electrode bank capturing a positive sputum sample, with both bacteria (in circles) and sputum debris (in squares) visible. **Bottom panel:** Bacteria isolated from medium only.

A real-time flow image of MTB capture by the DEP electrodes is presented in Supplemental Video S1. This shows the flow of solubilized sputum through the microfluidic chamber passing over the primary electrode bed and gradual trapping of bacilli onto the edges of the DEP electrodes and accumulating on the edges of subsequent electrodes from right to left as more sample is processed. The particle sizes vary; the smaller particles are captured bacilli (Figure 3). After saturation of the electrodes, the electrical field is switched off and the bacteria are immediately released, with a few remaining particles on the electrodes.

**Supplemental Figure S1:**
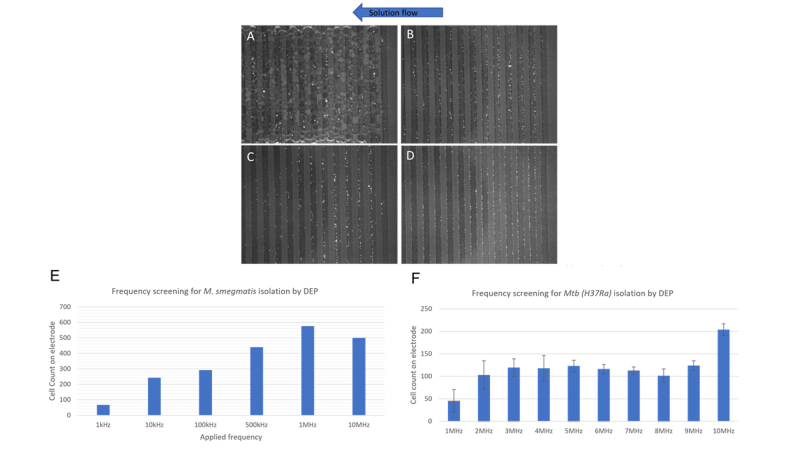
*Mycobacterium smegmatis* capture was noted from frequencies of 1 kHz. **A:** However, bubbles were quickly produced by the electrodes as a result of excessive joule heating, resulting in boiling of the solution. The number of bacterial cells captured increased in line with frequency up to 1 MHz. **B** and **C:** Visual analysis of capture highlighted that at <500 kHz, capture is not resulting from dielectrophoresis (DEP) but rather is a product of a.c. electroosmosis,^27^^,^^28^ with cells suspended along the center line of electrodes, appearing offset in the direction of solution flow, that is from right to left in this case. Such an effect increases the amount of irreversible cell adhesion to electrodes and therefore is not optimal for the purposes of bacterial isolation, which requires efficient release on field deactivation and is often associated with damage to the electrode surface with prolonged field activation. At 100 kHz, cells continue to concentrate via a.c. electroosmosis. **D:** DEP capture of bacterial cells is visualized as fluorescent bacterial particles accumulating at the edges of the electrodes only. **E:** Maxima counts from post-capture images following activation of electrodes at varying frequency are shown. **F:** As the DEP response of a target organism is inextricably linked to its individual cellular dimensions and composition, fine optimization of capture conditions was undertaken using *Mycobacterium tuberculosis* (MTB; H37Ra), and capture efficiency was found to be 10 MHz and was therefore selected for continued protocol development for MTB.

Initial experiments to optimize the DEP-mediated bacterial cell isolation were performed with *M. smegmatis*, suspended in 0.001× phosphate-buffered saline solution as a biosafe alternative to MTB. To determine the required optimal capture conditions, a.c. signals of varying frequency were applied to the cells, and their response to the field was monitored visually. The position of the cells on the electrodes was noted along with any additional phenomena observed, and representative images are presented in Supplemental Figure S1, A–D,^27^^,^^28^ along with maxima counts (Supplemental Figure S1, E and F), indicating the suitability of varying frequency currents for the isolation of *Mycobacteria*. The capture efficiency was noted to be highest at 10 MHz and at 10 Vpp. These conditions were therefore selected for continued protocol development for MTB.

### Sputum Sample Preparation Optimization

**Figure 4:**
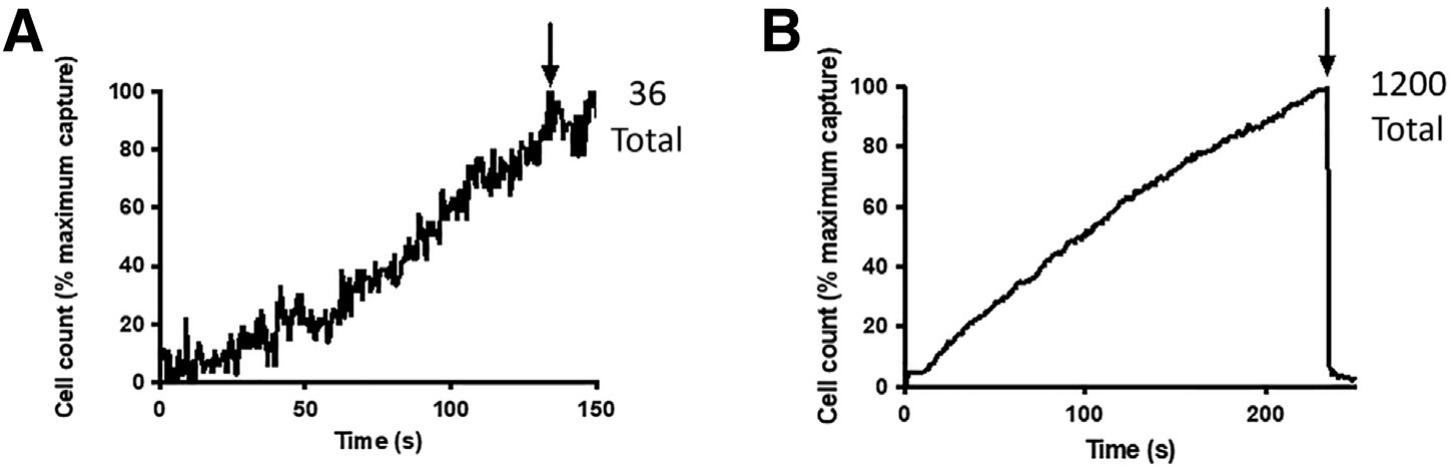
Particle counts before (**A**) and after (**B**) *Mycobacterium tuberculosis*–spiked sputum thinning optimization. Pre-optimization was with N-acetyl-l-cysteine (NALC) only. Postoptimization was with the lysis buffer described herein, with a 2-hour incubation at 40°C. **Arrows** indicate the time point at which the dielectric field was terminated and bacteria were released. Signal noise due to low capture numbers in the NALC-only run (**A**).

To apply CAPTURE-XT technology as a TB diagnostic tool, sputum samples must be compatible with the microfluidics without introducing chemicals or compounds that would affect the response of the bacterial cells to the DEP forces. As such, the commonly used mucolytic agents, such as N-acetyl-l-cysteine and sodium hydroxide, cannot be applied because of the salt concentration these chemicals contain. Initial optimization of flow rate, field strength, time of processing, and solute constituents were investigated using TB-negative human sputum spiked with cultured MTB bacilli or *M. smegmatis*. Using fluorescent microscopy and quantitative image software, the efficiency of capture and release was investigated. Of the compounds screened, dithiothreitol showed a consistent efficacy at reducing the viscosity of sputum that was enhanced by incubation at slightly elevated temperatures (40°C) and by the addition of detergents. Sputum optimal thinning required the addition of 2% dithiothreitol, 1% Tween 80, 1% Triton X-100, and 20 mmol/L EDTA in a 1:2 dilution with 5 minutes vortex and 120 minutes incubation at 40°C. Figure 4 demonstrates the improvements in capture and release with this protocol. The N-acetyl-l-cysteine thinning method resulted in a maximum capture of 36 bacteria over 150 seconds and did not release because of the viscosity. The optimized thinning method, however, captured >1200 bacteria in a 250-second period (876 in 150 seconds) and release was 97%.

Control of the ionic content of the processed sample is also required as the response of the targets to the DEP field is affected. This effect was reduced by dilution with de-ionized water. The number of cells captured by DEP was negligible in undiluted samples but was evident from a 1:5 dilution and essentially complete at 1:50 dilution with no cells noted traversing the electrodes without capture. Although 100% capture would be the goal, the capture efficiency at 1:25 dilution was optimal to minimize run times through the microfluidic device at linear flow velocity of 25 to 35 μL/minute. An optimized set of conditions was thus established before testing with clinical sputum specimens from TB cases and controls defined by conventional microbiological gold standard methods.

### Confirmatory PCR Assay Design

Following a positive visual diagnostic, indicated by the presence of bacteria on the electrodes, molecular analysis was used to confirm the presence of MTB bacilli as opposed to other organisms possibly present in the processed sputum. For this study, a qPCR assay was developed for three separate gene targets considered specific for MTB: *IS6110*, *IS1081*, and a bioinformatically determined MTB-specific target, *Rv1707*. Primer-probe combinations worked well as either individual assays or a triple-plex assay with limits of detection of <10 bacteria (calculated as genome copies equivalence from added DNA). Primer sets were screened for cross-reactivity against a panel of organisms, including most of the known MTB complex, a wide range of slow-growing mycobacteria, and known oral cavity or sputa bacterial contaminants. No significant cross-reactivity was noted *in silico* during the primer design stage. All primers displayed good species specificity for MTB when tested on purified DNA in PCR assays. Cross-reactivity in PCR assays with the panel of control organisms was assessed electrophoretically, with no-cross reactivity except for *Mycobacterium celatum*, which reacts weakly with *IS6110* and *IS1081*, and *Mycobacterium vaccae*, which also showed weak cross-reaction with *IS1081*. The results of species specificity testing are summarized in Supplemental Table S1. For the initial evaluation of DEP purification with a human sample panel, the *IS6110* primer-probe set was selected for use.

### CAPTURE-XT Evaluation with Sputa from TB Cases

#### Initial Validation Set

Protocol optimization and initial evaluation of the CAPTURE-XT technology and workflow were performed using biobanked sputum samples. Samples had been previously characterized using gold standard TB diagnostics, including culture and smear microscopy. Analysis of these characterized samples using the DEP isolation method was completed with both a visual result, in the form of a microscopy image of the collection electrodes, and a molecular readout from qPCR of the eluates. The 50 unblinded samples were for optimization of the protocol to be used on the 100 blinded sample set, as well as to demonstrate efficacy. To perform quantitative analyses on the optimization set, a data set with controlled variables was pulled out using the following exclusion criteria.

• All samples must be run on the same chip type.
• All samples must be thinned with the same thinning solution.
• All samples must correlate with the known burden (eg, if there was capture with a negative, or no capture with a 3+).
• All samples ran without the appearance of bubble formation from electrolysis effects or surface coat defects.

After establishing these initial methodological variables, 17 sputum samples were used with the selected method to assess performance ahead of the blinded study. These were four smear negatives and six smear negative and culture positive: two 1+, two 2+, and three 3+ (Tables 2 and 3). Note that FIND defined smear negative and culture positive as scanty, whereas the usual definition of scanty is smear positive/culture positive. For these unblinded samples, the authors are examining smear negative/culture positive.

**Table 2:** qPCR C_T_ Values for Unblinded Sputum Samples Graded Bacteriologically

Smear status	Mean C_T_	SD	*N*
Negative	38.4	1.1	4
S–C+	35.7	1.8	6
1+	27.5	1.0	2
2+	26.6	2.3	2
3+	24.2	2.0	3

*N*, number of samples included; Negative, smear and culture negative; qPCR, real-time quantitative PCR; S–C+, smear negative and culture positive.

**Table 3:** Unblinded Evaluation of Test Set of Sputa

FIND smear result	Samples tested	DEP positive	PCR positive	Concordance with gold standard, %	
				DEP	PCR
3+	3	3	3	100	100
2+	2	2	2	100	100
1+	2	2	2	100	100
S–C+	6	6	6	100	100
Negative	4	0∗	2†	100	50

DEP, dielectrophoresis; FIND, Foundation for Innovative New Diagnostics; S–C+, smear negative and culture positive.

∗ Two negatives that had immediate, obvious capture were mislabeled and therefore excluded.

† C_T_ values were 37.7 and 37.6, which are borderline positive by the set criteria.

**Figure 5:**
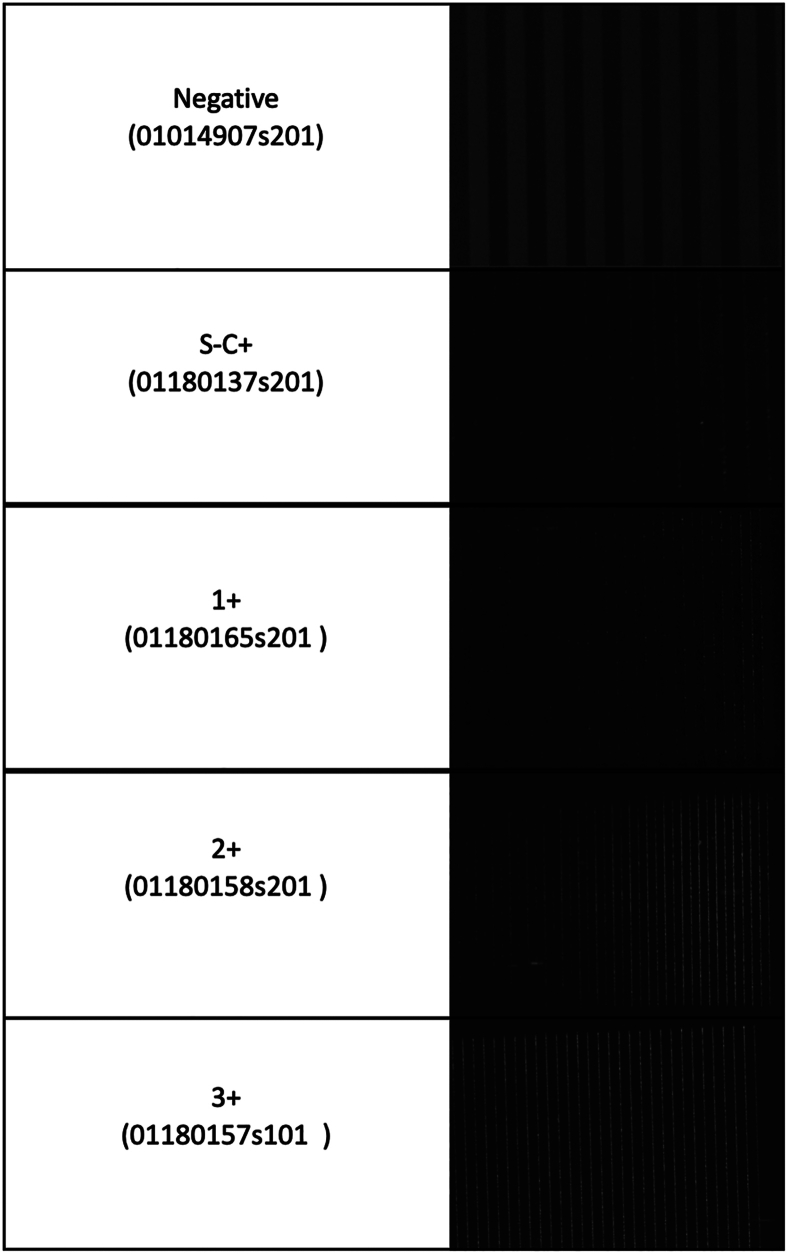
Representative samples from each of the smear microscopy and culture classifications of tuberculosis burden are presented with sample identification number in parentheses. Bacilli were clearly visible at the edges of electrodes, where the field strength is greatest, indicating successful cell capture from the sputum sample. *Mycobacterium tuberculosis* (MTB) smear-negative and culture-positive (S–C+) sputum showed only minimal captured bacteria. All samples demonstrated a low level of background non-specific captured debris. A correlation between smear-positive grading and visual count of captured particles interpreted as MTB bacilli was seen. Correlation between MTB DNA quantification by species-specific real-time quantitative PCR was also seen with the degree of captured fluorescent particles on the dielectrophoresis electrodes (Table 2). Images of negative and S–C+ samples are magnified (×100, rather than ×40) for better clarity.

Positive qPCR amplification of the *IS6110* target using sample eluates showed C_T_ values in approximate proportion to both the smear microscopy grade and the visual bacterial accumulation on the DEP electrodes (Figure 5). There was a clear proportionality between C_T_ value and bacteriological load, estimated by smear status. The overall qPCR data for these smear-graded unblinded samples for a total of 17 samples are shown in Table 2. This semiquantitative correlation between bacteria load and genome equivalents measured by qPCR confirms the potential suitability of this bacterial capture and purification method for use ahead of molecular diagnostic tests.

Initial concordance between gold standard diagnostic criteria and the combined visual imaging/qPCR data for these 17 selected samples (above) is presented in Table 3. Good concordance was evident for the smear-positive samples, graded from smear negative/culture positive to 3+.

Two of the negative samples were strongly positive in the DEP run and were therefore excluded as they had, on examination by FIND, been mislabeled (Table 3). Concordance with qPCR for the smear-negative samples was 50% due to the qPCR results being borderline positive (C_T_, 37.7 and 37.6) and therefore did not reach the negative cutoff C_T_ (38) (Table 3). However, these initial experiments demonstrate a proof of principle that DEP capture and visualization, and quantitation by qPCR, shows concordance and a proportionality for the graded smear-positive sputum samples.

MTB confirmatory qPCR assays showed a significant difference between the thinned sputum samples' C_T_ values and the post-DEP eluate samples. The average decrease in C_T_ from thinned sputum sample to post-DEP eluate for scanty, 1+, 2+, and 3+ sets was 0.8 (*P* = 0.02), 0.9 (*P* = 0.003), 1.34 (*P* = 0.02), and 0.9 (*P* = 0.007), respectively. This equates to a fold change of 1.7, 1.9, 2.5, and 1.9, respectively. These data indicate that the bacterial particles visualized as being captured by DEP and then released are MTB because there is enrichment with MTB in the eluate compared with noncaptured thinned sputum samples.

#### Blinded Sputum Set Analysis

As the results from the unblinded samples had shown good correlation with the known bacteriological status of the samples, the developed method described was subsequently used for the analysis of a 100-sample set, provided without clinical information, allowing a blind assessment of the technology against gold standard diagnostics.

The 100 blinded sputum samples were processed using the optimized protocol. Images from visual analysis electrode arrays were captured at the end of each sample run and used to make the primary diagnostic call in a binary manner as MTB positive or MTB negative. Eluates were collected following imaging for molecular analysis with *IS6110* qPCR. A sample with a C_T_ of <38 was considered positive; however, samples returning a C_T_ value between 35 and 38 were considered borderline. In this case, the qPCR data were examined, and a judgement of positivity was made and agreed by laboratory staff (ie, shape of amplification curve and apparent titration of diluted samples). Where samples were discrepant between the visual DEP and PCR result, such samples were classified as positive. Of the 100 samples tested, 66 were classified as positive, 27 were classified as negative, and 7 were classified as borderline. Results were submitted to FIND for unblinding, and full clinical data were returned with concordance determination. An overall concordance of 86% was seen across positive samples, with 100%, 90%, and 84% for 3+, 2+, and 1+ smear classifications, respectively. Concordance was lower for smear-negative samples, with 76% agreement between gold standard and the CAPTURE-XT method.

#### Discordance Testing

To resolve the discordant samples, second aliquots from the same initial sputa samples were subject to repeated analysis. Discordance analysis was completed on a second iteration of the CAPTURE-XT platform and higher-capacity fluidic consumable in line with ongoing technology developments. To ensure sensitivity was maximized, the full 500-μL sputum sample was processed, rather than running to visual positivity. To improve qPCR specificity, the *IS6110* PCR was combined with *IS1081* and the *Rv1707* gene targets in a multiplex format. Completion of the repeated analysis returned improved results with an overall concordance of 93% for smear-positive samples with three smear scanty samples remaining false negatives. The concordance of smear-negative/culture-positive samples remained at 76.9%. The triplex PCR enabled improved discrimination of amplification from PCR artifacts and reduced false-positive calls from 15 to 1. However, on further investigation, this remaining one false-positive sample that was classified originally as smear and culture negative returned positive after prolonged bacteriological culture in the authors’ laboratory, confirming the positive qPCR result. Overall, the DEP purification method returned a concordance of 76.9% for smear-negative, culture-positive samples, with an overall concordance for all culture positives of 87%. The sensitivity and specificity were 87% ± 16.9% and 100% ± 19.6%, respectively. The final results of the study are presented in Table 4.

**Table 4:** Final 100-Sample Test Performance Evaluation

Sample classification	Samples tested	Correct diagnosis	Concordance, %
Total	100		
MTB negative	29	29	100
MTB positive	71	62	87
Smear negative/culture positive (total)	26	20	76.9
Smear positive (total)	45	42	93
Scanty/1+	25	22	88
2+	10	10	100
3+	10	10	100

Number of samples with classification by Foundation for Innovative New Diagnostics (FIND). MTB-positive samples subdivided by smear status and rating. Concordance calculated as percentage of samples for which the experimental protocol returned the same diagnostic outcome (MTB positive or MTB negative) as the FIND gold standard diagnostics.

MTB, *Mycobacterium tuberculosis*.

## Discussion

The eradication of TB, as one of the Sustainable Development Goals set by the United Nations, requires not only tests with improved sensitivity but those with the potential to be used in near-patient settings, beyond the confines of laboratories and health care centers, where vast numbers of TB-infected individuals require them.^10^^,^^12^ The technology presented in this study demonstrates the potential of CAPTURE-XT for the capture, release, and subsequent detection by qPCR of MTB from sputum samples. Overall, the prototype CAPTURE-XT platform achieved a specificity of 100% and a sensitivity of 87%. In comparison, GeneXpert has been shown to have a specificity of 90.4% and a sensitivity of 78.2%; and Ultra has a specificity of 98.7% and a sensitivity of 87.5%.^11^^,^^29^ Concordance rates between gold standard diagnostics and DEP purification with MTB-specific qPCR assays were 100% for 2+ and 3+ smear-positive samples. Lower bacterial burden samples (smear negative, culture positive), which are more likely to give false-negative results in standard diagnostic tests, had a concordance of 76.9% in the DEP/qPCR assay.^30^ Interpretation of qPCR was improved when three targets were used. This could be due to the variable copy number of *IS6110*, which can be present in 1 to 20 copies or absent, depending on strain, whereas *IS1081* and *Rv1707* are present in stable copy number of 6 and 1, respectively, producing a more reliable pan-strain test.^31^ The DEP/qPCR study described here used species-specific qPCR as a cross-validating assay to show efficiency of capture, purification, and release of MTB by DEP directly from solubilized sputum specimens. Also, the C_T_ value cutoff used in this proof-of-principle study was 38. This chosen negative threshold value (C_T_ = 38) and the subjective decisions as to positivity for some of the low bacterial burden samples with C_T_ = 35 to 38 is a limitation of this study, which had insufficient samples to determine a more quantitative estimation. Although there is little consensus on what the cutoff value should be across different tests, the C_T_ value cutoff for GeneXpert is 38, which was why that value was chosen for this study. In addition, some samples that were deemed positive in the DEP system were, on further inspection by FIND, positives and had been erroneously labeled as negatives. This system, therefore, was able to detect MTB bacteria that had previously gone undiagnosed. In fact, the qPCR result for several samples collected from thinned sputum only were negative, but post-DEP eluates were positive because of the concentrating effect of DEP, which means samples with low burden are less likely to be counted as false negatives. Once optimized, this system could, therefore, have an increased sensitivity.

In this feasibility stage, all work was performed in a biosafety cabinet, with connecting tubing, which resulted in a large dead volume. These issues affect the speed and accessibility of the assay and reduced the potential DEP concentration effect through sample dilution. However, condensing the system into a portable front-end device would resolve these issues. The sample study presented here was limited to processing 500 μL of sputum. For improved sensitivity and an expedited time to result, devices with highly paralleled capture channels are being developed to increase throughput. Current microfluidic setup can capture the bacteria in a sample in <1 hour with a visual readout for an initial diagnosis before qPCR analysis for low-burden samples and for drug resistance profiling. Refinements to aspects of the biological processing and device design are anticipated to result in significantly improved performance. For this study, biobanked samples were gratefully utilized; however, prior storage at −80°C may have resulted in a loss of cell viability or affected the bacterial cell membrane required to elicit a response to dielectrophoresis. Repeated freeze-thawing reduced the viability of the samples in our hands (data not shown).^32^ Furthermore, the effect of freezing must be considered with regard to the ease with which thinning can be achieved for microfluidic compatibility. To advance the performance analysis of this technology, fresh samples are required to better simulate a real-world scenario for point-of-care diagnostics. The fluorescent dye employed in this study was not specific for MTB and therefore could have given false positives in a mixed sample. Using an MTB-specific dye would improve the specificity of the visual analysis aspect of this technology.^33^ This assay may also be used in the future for stratification of treatment.

In summary, we have demonstrated, as a proof of principle using biobanked TB sputum specimens and an experimental DEP capture technology, that CAPTURE-XT is a sensitive and specific platform for TB diagnosis. This has potential as a next-generation TB diagnostic or as a front-end sample preparation technology for visual and subsequent molecular detection techniques. In addition, the qPCR assay could easily include detection of markers for drug resistance, making this technology capable of personalized medicine.

## Supplemental Data

Supplemental Table S1

Supplemental Video S1 Video of capture and release from the primary electrodes. The camera is paused once the electrodes are saturated, and then restarted at *t* = 45 seconds to record the release.

## References

[1] Walzl G., McNerney R., du Plessis N., Bates M., McHugh T.D., Chegou N.N., Zumla A. (2018). Tuberculosis: advances and challenges in development of new diagnostics and biomarkers. Lancet Infect Dis.

[2] World Health Organization: Global Tuberculosis Report 2019. Geneva, Switzerland: World Health Organization, 2019. Available at: https://www.who.int/publications/i/item/9789241565714 (accessed January 26, 2021)

[3] Dzodanu E.G., Afrifa J., Acheampong D.O., Dadzie I. (2019). Diagnostic yield of fluorescence and Ziehl-Neelsen staining techniques in the diagnosis of pulmonary tuberculosis: a comparative study in a district health facility. Tuberc Res Treat.

[4] Lombardi G., Di Gregori V., Girometti N., Tadolini M., Bisognin F., Dal Monte P. (2017). Diagnosis of smear-negative tuberculosis is greatly improved by Xpert MTB/RIF. PLoS One.

[5] Singhal R., Myneedu V.P. (2015). Microscopy as a diagnostic tool in pulmonary tuberculosis. Int J Mycobacteriol.

[6] Steingart K.R., Henry M., Ng V., Hopewell P.C., Ramsay A., Cunningham J., Urbanczik R., Perkins M., Aziz M.A., Pai M. (2006). Fluorescence versus conventional sputum smear microscopy for tuberculosis: a systematic review. Lancet Infect Dis.

[7] World Health Organization: Improving the diagnosis and treatment of smear-negative pulmonary and extrapulmonary tuberculosis among adults and adolescents: Recommendations for HIV-prevalent and resource-constrained settings. Geneva, Switzerland: World Health Organization, 2007. Available at: https://apps.who.int/iris/handle/10665/69463 (accessed May 11, 2023)

[8] Grandjean L., Martin L., Gilman R.H., Valencia T., Herrera B., Quino W., Ramos E., Rivero M., Montoya R., Escombe A.R., Coleman D., Mitchison D., Evans C.A. (2008). Tuberculosis diagnosis and multidrug resistance testing by direct sputum culture in selective broth without decontamination or centrifugation. J Clin Microbiol.

[9] Balcha T.T., Sturegård E., Winqvist N., Skogmar S., Reepalu A., Jemal Z.H., Tibesso G., Schön T., Björkman P. (2014). Intensified tuberculosis case-finding in HIV-positive adults managed at Ethiopian health centers: diagnostic yield of xpert MTB/RIF compared with smear microscopy and liquid culture. PLoS One.

[10] Falzon D., Migliori G.B., Jaramillo E., Weyer K., Joos G., Raviglione M. (2017). Digital health to end tuberculosis in the sustainable development goals era: achievements, evidence and future perspectives. Eur Respir J.

[11] Mechal Y., Benaissa E., El Mrimar N., Benlahlou Y., Bssaibis F., Zegmout A., Chadli M., Malik Y.S., Touil N., Abid A., Maleb A., Elouennass M. (2019). Evaluation of GeneXpert MTB/RIF system performances in the diagnosis of extrapulmonary tuberculosis. BMC Infect Dis.

[12] World Health Organization: Global tuberculosis report 2020. Geneva, Switzerland: World Health Organization, 2020. Available at: https://www.who.int/publications/i/item/9789240013131 (accessed January 26, 2021)

[13] World Health Organization (2017). WHO meeting report of a technical expert consultation: non-inferiority analysis of Xpert MTF/RIF Ultracompared to Xpert MTB/RIF.

[14] World Health Organization: Global tuberculosis report 2018. Geneva, Switzerland: World Health Organization, 2018. Available at: https://apps.who.int/iris/handle/10665/274453 (accessed January 26, 2021)

[15] Seki M., Kim C.K., Hayakawa S., Mitarai S. (2018). Recent advances in tuberculosis diagnostics in resource-limited settings. Eur J Clin Microbiol Infect Dis.

[16] Van Rie A., Page-Shipp L., Scott L., Sanne I., Stevens W. (2010). Xpert MTB/RIF for point-of-care diagnosis of TB in high-HIV burden, resource-limited countries: hype or hope?. Expert Rev Mol Diagn.

[17] Hawkins B.G., Lai N., Clague D.S. (2020). High-sensitivity in dielectrophoresis separations. Micromachines (Basel).

[18] Fernandez R.E., Rohani A., Farmehini V., Swami N.S. (2017). Review: microbial analysis in dielectrophoretic microfluidic systems. Anal Chim Acta.

[19] Hughes M.P. (2016). Fifty years of dielectrophoretic cell separation technology. Biomicrofluidics.

[20] Yang L. (2012). A review of multifunctions of dielectrophoresis in biosensors and biochips for bacteria detection. Anal Lett.

[21] Global Laboratory Initiative: Mycobacteriology Laboratory Manual. Stop TB Partnership, 2014. Available at: https://stoptb.org/wg/gli/assets/documents/gli_mycobacteriology_lab_manual_web.pdf (accessed May 11, 2023)

[22] Pohl H.A., Hawk I. (1966). Separation of living and dead cells by dielectrophoresis. Science.

[23] Pohl H.A., Crane J.S. (1971). Dielectrophoresis of cells. Biophys J.

[24] Schindelin J., Arganda-Carreras I., Frise E., Kaynig V., Longair M., Pietzsch T., Preibisch S., Rueden C., Saalfeld S., Schmid B., Tinevez J.-Y., White D.J., Hartenstein V., Eliceiri K., Tomancak P., Cardona A. (2012). Fiji: an open-source platform for biological-image analysis. Nat Methods.

[25] Edelstein A.D., Tsuchida M.A., Amodaj N., Pinkard H., Vale R.D., Stuurman N. (2014). Advanced methods of microscope control using μManager software. J Biol Methods.

[26] Untergasser A., Nijveen H., Rao X., Bisseling T., Geurts R., Leunissen J.A.M. (2007). Primer3Plus, an enhanced web interface to Primer3. Nucleic Acids Res.

[27] Islam N., Wu J. (2006). Microfluidic transport by AC electroosmosis. J Phys Conf Ser.

[28] Oh J., Hart R., Capurro J., Noh H. (2009). Comprehensive analysis of particle motion under non-uniform AC electric fields in a microchannel. Lab Chip.

[29] Chakravorty S., Simmons A.M., Rowneki M., Parmar H., Cao Y., Ryan J., Banada P.P., Deshpande S., Shenai S., Gall A., Glass J., Krieswirth B., Schumacher S.G., Nabeta P., Tukvadze N., Rodrigues C., Skrahina A., Tagliani E., Cirillo D.M., Davidow A., Denkinger C.M., Persing D., Kwiatkowski R., Jones M., Alland D. (2017). The new Xpert MTB/RIF ultra: improving detection of Mycobacterium tuberculosis and resistance to rifampin in an assay suitable for point-of-care testing. MBio.

[30] Rieder H.L., Lauritsen J.M., Naranbat N., Katamba A., Laticevschi D., Mabaera B. (2009). Quantitative differences in sputum smear microscopy results for acid-fast bacilli by age and sex in four countries. Int J Tuberc Lung Dis.

[31] Alonso H., Samper S., Martín C., Otal I. (2013). Mapping IS6110 in high-copy number Mycobacterium tuberculosis strains shows specific insertion points in the Beijing genotype. BMC Genomics.

[32] Shu Z., Weigel K.M., Soelberg S.D., Lakey A., Cangelosi G.A., Lee K.H., Chung J.H., Gao D. (2012). Cryopreservation of mycobacterium tuberculosis complex cells. J Clin Microbiol.

[33] Kamariza M., Shieh P., Ealand C.S., Peters J.S., Chu B., Rodriguez-Rivera F.P., Babu Sait M.R., Treuren W.V., Martinson N., Kalscheuer R., Kana B.D., Bertozzi C.R. (2018). Rapid detection of Mycobacterium tuberculosis in sputum with a solvatochromic trehalose probe. Sci Transl Med.

