# Supplementary material for: A Novel Microfluidic Dielectrophoresis Technology to Enable Rapid Diagnosis of Mycobacteria tuberculosis in Clinical Samples
Source: J Mol Diagn. 2023 Jun 22;25(7):513–23. doi: 10.1016/j.jmoldx.2023.04.005 (PMC12179504; doi:10.1016/j.jmoldx.2023.04.005)
Supplement: Supplemental Table S1 [file mmc1.docx]

**Supplementary Table 1**: Summary of species-specificity of the newly designed TaqMan primer pairs using the exclusivity panel of organisms. *🗶 = no cross-reactivity ✓ = correct specificity*

|  | *RV1707*  (134bp) | IS6110  (135bp) | *IS1081*  (138bp) |
| --- | --- | --- | --- |
| M. tuberculosis complex |  |  |  |
| *M. tuberculosis (H37Rv)* | ✓ | ✓ | ✓ |
| *M. bovis (BCG)* | ✓ | ✓ | ✓ |
| *M. bovis* | ✓ | ✓ | ✓ |
| *M. africanum* | ✓ | ✓ | ✓ |
| *M. canetti* | ✓ | ✓ | ✓ |
| *M. microti* | ✓ | ✓ | ✓ |
| Slow-growing mycobacteria |  |  |  |
| *M. asiaticum* | Faint band | 🗶 | 🗶 |
| *M. avium subspecies avium (S4)* | 🗶 | 🗶 | 🗶 |
| *M. celatum* | 🗶 | ✓ | ✓ |
| *M. chimaera* | 🗶 | 🗶 | 🗶 |
| *M. gastri* | 🗶 | 🗶 | 🗶 |
| *M. gordonae* | 🗶 | 🗶 | 🗶 |
| *M. haemophilum* | 🗶 | 🗶 | 🗶 |
| *M. intracellulare (Type 7)* | 🗶 | 🗶 | 🗶 |
| *M. interjectum* | 🗶 | 🗶 | 🗶 |
| *M. kansasii* | 🗶 | 🗶 | 🗶 |
| *M. lentiflavum(Strain 1)* | 🗶 | 🗶 | 🗶 |
| *M. lentiflavum(Strain 2)* | 🗶 | 🗶 | 🗶 |
| *M. marinum* | 🗶 | 🗶 | 🗶 |
| *M. malmoense* | 🗶 | 🗶 | 🗶 |
| *M. scrofulaceum* | 🗶 | 🗶 | 🗶 |
| *M. shimoidei* | 🗶 | 🗶 | 🗶 |
| *M. simae* | 🗶 | 🗶 | 🗶 |
| *M. szulgai* | 🗶 | 🗶 | 🗶 |
| *M. terrae (Strain 1)* | 🗶 | 🗶 | 🗶 |
| *M. terrae (Strain 2)* | 🗶 | 🗶 | 🗶 |
| *M. ulcerans (strain 912)* | 🗶 | 🗶 | 🗶 |
| *M. xenopi* | 🗶 | 🗶 | 🗶 |
| Fast-growing mycobacteria |  |  |  |
| *M. abscessus* | 🗶 | 🗶 | 🗶 |
| *M. chelonae* | 🗶 | 🗶 | 🗶 |
| *M. flavescens* | 🗶 | 🗶 | 🗶 |
| *M. fortuitum* | 🗶 | 🗶 | 🗶 |
| *M. perigrinum* | 🗶 | 🗶 | 🗶 |
| *M. phlei* | 🗶 | Faint band | 🗶 |
| *M. porcinum* | 🗶 | 🗶 | 🗶 |
| *M. septicum* | 🗶 | 🗶 | 🗶 |
| *M. smegmatis MC^2^155* | 🗶 | 🗶 | 🗶 |
| *M. vaccae* | 🗶 | 🗶 | ✓ |
|  |  |  |  |
| Other bacteria potentially present in sputum: |  |  |  |
| *Acinetobacter baumannii* | 🗶 | 🗶 | 🗶 |
| *Citrobacter freundii* | 🗶 | 🗶 | 🗶 |
| *Corynebacterium striatum* | 🗶 | 🗶 | 🗶 |
| *Enterobacter cloacae* | 🗶 | 🗶 | 🗶 |
| *Escherichia coli* | 🗶 | 🗶 | 🗶 |
| *Hemophilus influenzae* | Faint band | ✓ | 🗶 |
| *Klebsiella pneumoniae* | 🗶 | 🗶 | 🗶 |
| *Moraxella catarrhalis* | 🗶 | 🗶 | 🗶 |
| *Pseudomonas aeruginosa* | 🗶 | 🗶 | 🗶 |
| *Staphylococcus aureus* | 🗶 | 🗶 | 🗶 |
| *Streptococcus agalactiae* | 🗶 | Faint band | 🗶 |
| *Streptococcus agalactiae( strain2)* | 🗶 | 🗶 | 🗶 |
| *Streptococcus mitis* | 🗶 | 🗶 | 🗶 |
| *Streptococcus pneumoniae* | 🗶 | 🗶 | 🗶 |
| *Streptococcus pyogenes* | 🗶 | 🗶 | 🗶 |

1ng, which is equivalent to 10^6^ bacteria, was used as input DNA for the PCR and visual inspection for correct *Mtb*-specific amplicon size was used as the electrophoretic readout. It is important to note a more clinically relevant sample input would have, at most, 10^3^ to 10^4^ with mixed infections. qPCR was carried out on all 52 samples at both 1ng, and 0.01ng input DNA (results not shown here). The only strains that gave amplification at a clinically relevant sample input (0.01ng) were the TB complex strains. None of the other strains showed cross reactivity.
